# Exhaled Breath Reflects Prolonged Exercise and Statin Use during a Field Campaign

**DOI:** 10.3390/metabo11040192

**Published:** 2021-03-24

**Authors:** Ben Henderson, Guilherme Lopes Batista, Carlo G. Bertinetto, Joris Meurs, Dušan Materić, Coen C. W. G. Bongers, Neeltje A. E. Allard, Thijs M. H. Eijsvogels, Rupert Holzinger, Frans J. M. Harren, Jeroen J. Jansen, Maria T. E. Hopman, Simona M. Cristescu

**Affiliations:** 1 Department of Molecular and Laser Physics, Institute for Molecules and Materials, Radboud University, 6525 XZ Nijmegen, The Netherlands; b.henderson@science.ru.nl (B.H.); G.LopesBatista@science.ru.nl (G.L.B.); joris.meurs@ru.nl (J.M.); d.materic@uu.nl (D.M.); F.Harren@science.ru.nl (F.J.M.H.); 2 Department of Analytical Chemistry and Chemometrics, Institute for Molecules and Materials, Radboud University, 6525 XZ Nijmegen, The Netherlands; c.bertinetto@science.ru.nl (C.G.B.); jj.jansen@science.ru.nl (J.J.J.); 3 Institute for Marine and Atmospheric Research, Utrecht University, 3584 CC Utrecht, The Netherlands; r.holzinger@uu.nl; 4 Department of Physiology, Radboud Institute for Health Sciences, Radboud University Medical Center, 6525 XZ Nijmegen, The Netherlands; Coen.Bongers@radboudumc.nl (C.C.W.G.B.); Eline.Allard@radboudumc.nl (N.A.E.A.); Thijs.Eijsvogels@radboudumc.nl (T.M.H.E.); Maria.Hopman@radboudumc.nl (M.T.E.H.)

**Keywords:** prolonged exercise, breath, short-chain fatty acids, statin, PTR-ToF-MS

## Abstract

Volatile organic compounds (VOCs) in exhaled breath provide insights into various metabolic processes and can be used to monitor physiological response to exercise and medication. We integrated and validated in situ a sampling and analysis protocol using proton transfer reaction time-of-flight mass spectrometry (PTR-ToF-MS) for exhaled breath research. The approach was demonstrated on a participant cohort comprising users of the cholesterol-lowering drug statins and non-statin users during a field campaign of three days of prolonged and repeated exercise, with no restrictions on food or drink consumption. The effect of prolonged exercise was reflected in the exhaled breath of participants, and relevant VOCs were identified. Most of the VOCs, such as acetone, showed an increase in concentration after the first day of walking and subsequent decrease towards baseline levels prior to walking on the second day. A cluster of short-chain fatty acids including acetic acid, butanoic acid, and propionic acid were identified in exhaled breath as potential indicators of gut microbiota activity relating to exercise and drug use. We have provided novel information regarding the use of breathomics for non-invasive monitoring of changes in human metabolism and especially for the gut microbiome activity in relation to exercise and the use of medication, such as statins.

## 1. Introduction

Certain stimuli, such as exercise or medication, affect various biochemical and metabolic processes in the human body that in turn alter the composition and concentration of the associated volatile metabolites [1,2]. Analysis of these metabolites, particularly of volatile organic compounds (VOCs) in exhaled breath, provide a snapshot of the current health status and disease activity of an individual [3,4].

Proton-transfer reaction time of flight mass spectrometry (PTR-ToF-MS) has been successfully employed to exhaled breath research conducted in controlled laboratory environments or under clinical settings [5,6,7,8]. However, its versatility outside of this controlled environment regarding exhaled breath analysis is yet to be demonstrated.

The effect of exercise on breath VOCs has been previously investigated using proton transfer reaction mass spectrometry (PTR-MS). These studies typically targeted specific VOCs, such as acetone and isoprene during short-term, intense exercise on an ergometer [9,10,11,12]. The Four Days Marches is one of the biggest walking events in the world that attracts up to 50,000 participants every year to Nijmegen, the Netherlands, where participants undertake repeated and prolonged exercise in the form of walking either 30, 40, or 50 km at a self-selected pace on four consecutive days. Previously, we showed that breath acetone collected from participants during the Four Days Marches in Mylar bags and analyzed in the laboratory can be used to monitor the effects of prolonged and repeated exercise on whole body lipolysis and hepatic ketogenesis in field conditions [13].

This research will focus on investigating the effect of prolonged and repeated exercise over several days, using an online sampling protocol and considering the whole breath profile in real time, rather than a pre-selection of VOCs. The participant cohort is comprised of non-statin and statin users. Statins are a commonly prescribed medication to lower blood cholesterol levels [14] and subsequently reduce illness and mortality in those who are at high risk of cardiovascular events. Furthermore, 7–29% of statin users develop muscle complaints during treatment [14], which may intensify with exercise and cause variations in the physiological response between non-statin and statin users to prolonged and repeated exercise.

In this study, we validate and report a sampling and analysis protocol integrated into an in-situ approach that has been validated for online breath analysis using PTR-ToF-MS in a field campaign, without controlling any external factors, such as food and drinks consumption. Our goal was to investigate whether under these conditions, the effect of prolonged and repeated exercise is reflected in the breath VOCs profile, and in that case, we identify the significant VOCs, along with their variation over time. Finally, we propose to reveal the interaction between prolonged exercise and statin use in exhaled breath and highlight relevant metabolic distinctions.

## 2. Results

### 2.1. Assessment of Sampling Methodology

The participants included in this study were enrolled onto a multiple diagnostic testing pipeline with breath sampling being the first; therefore, a key challenge was to ensure that the sampling protocol was suitable for a high throughput of samples. An assessment of a real-time sampling methodology was carried out to determine (i) the time needed per participant to facilitate a high throughput of samples, (ii) the reproducibility of duplicate breath samples, and (iii) carryover effects from successive breath samples between participants.

**Figure 1:**
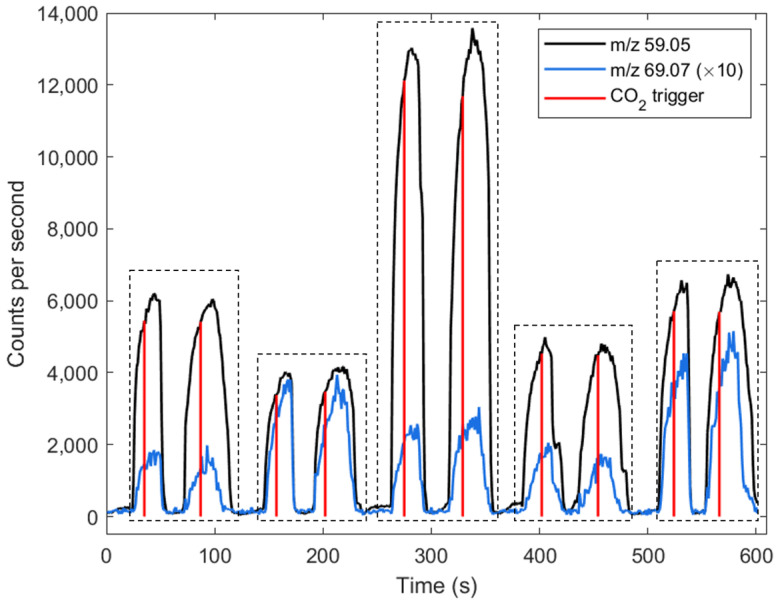
Raw signals of *m*/*z* 59.05 (acetone) and *m*/*z* 69.07 (isoprene) from five consecutive participants duplicate breath samples (each participant is separated using the dashed boxes). The red line represents the trigger that was activated when the CO_2_ concentration in exhaled breath reached 4%. On average, two minutes per participant are sufficient to collect the samples.

Using the online sampling method presented in this paper, it took approximately two minutes to collect two exhalations per participant. An example of the raw traces of acetone and isoprene of five participants measured consecutively, along with the CO_2_-flow monitoring trigger used for assessing the extraction quality of breath samples, are presented in Figure 1.

The intra-participant variation was assessed by calculating Lin’s concordance coefficient (*R_c_*) [15]. For all except one of the participants (*R_c_* = 0.84), the *R_c_* values exceeded 0.97, indicating an excellent repeatability between successive breath samples (Figure S1) [16]. This demonstrates that the on-line sampling method provided repeatable PTR-ToF-MS data acquisition for consecutive breath samples and subsequently robust data for further analysis.

Since no strict restrictions were placed on participants in regard to eating and drinking prior to breath sampling, abnormally high concentrations were occasionally recorded from some participants for compounds assigned to ethanol (from alcohol consumption) and monoterpenes (from e.g., gum, toothpaste, or beverage). An external flushing protocol of the breath sampling line was applied to eliminate the carry over effects between breath samples (Figure S2).

### 2.2. Effect of Prolonged Exercise on Breath Profile

Initially, multilevel principal component analysis (M-PCA) containing data from all time points (Day 0, Day 1, Day 2 am, Day 2, and Day 3) for all the three statin groups (non-statin users: St0; statin users: St1; statin users with muscle complaints: St2) was used as an exploratory tool to identify any patterns in the data. Breath profiles for Day 0 were separated from the other sample time points, which we propose is predominantly due to the effect of prolonged exercise. No particular clustering was seen for the statin groups and no outliers were observed (Figure S3).

**Figure 2:**
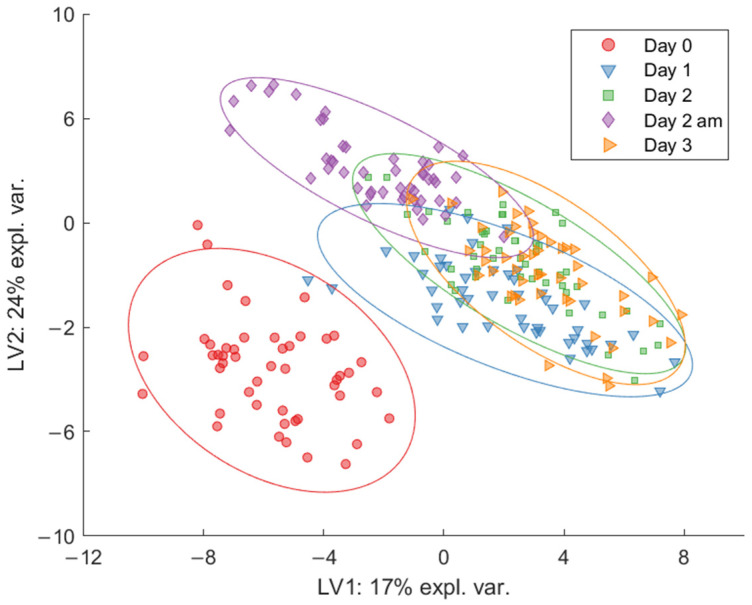
Multilevel partial least squares discriminant analysis (M-PLS-DA) model for the effect of walking on the breath volatile organic compound (VOC) profile for St0. The model includes all sample time points. A clear separation between Day 0 and the remaining time points (Day 1–Day 3) is revealed, indicating a significant change in the breath profile at the onset of exercise compared against Day 0.

To further investigate how the breath VOC profiles changed over time, multilevel partial least squares discriminant analysis (M-PLS-DA) was applied on the PTR-ToF-MS data for each of the three statin groups individually. A clear separation was found between Day 0 and the other time points, indicating that exercise is reflected on the breath VOCs profile of all participants. This exercise-induced effect was observed for St0 (Figure 2), St1, and St2 (Figure S4).

**Figure 3:**
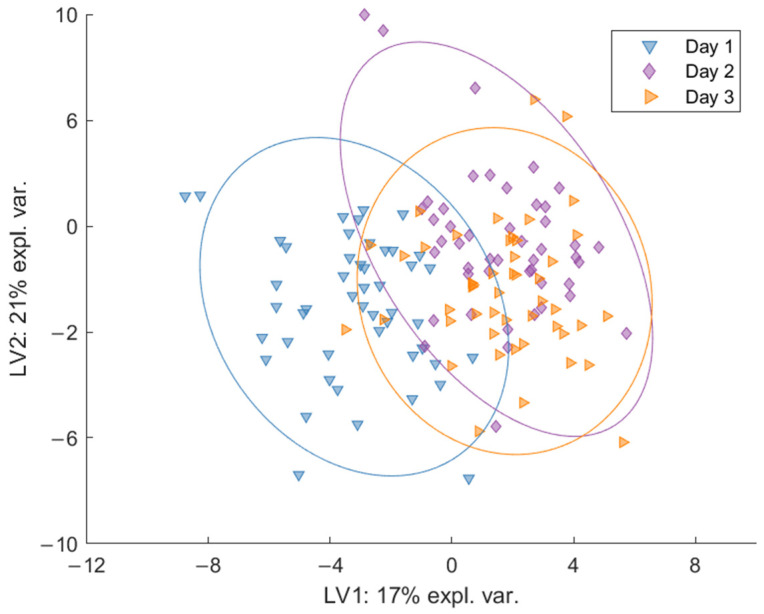
M-PLS-DA model for St0 for all post walking measurements (Day 1, Day 2, and Day 3) with a difference in clustering along the first latent variable (LV1). There is high overlap between Day 2 and Day 3 and a degree of separation between Day 1 and the other time points on LV1, suggesting a possible adaptation to prolonged exercise after the 2nd and 3rd day of walking.

The differences between the post-walking measurements were explored by additional local M-PLS-DA models considering only data from Day 1, Day 2, and Day 3 for each group separately. The trend observed for each group was similar (Figure 3 for St0 and Figure S5 for St1 and St2, respectively). Separation was observed between the breath profiles on Day 1 and Day 2 and Day 3 along the first latent variable (LV1). The breath profiles for Day 2 and Day 3 overlap, suggesting that Day 2 and Day 3 have similar breath profiles. This result suggests an acute response to the first day of walking followed by a progression toward a steady state, as the participants somewhat adapt to the prolonged and repeated exercise.

All ions with a Variable’s Importance in Projection (VIP) score >1 were deemed to reflect the effect of prolonged exercise, as resulted from each of the M-PLS-DA models, and these are reported in a Venn diagram (Figure S6). Endogenous compounds were assigned a putative molecular formula and name using accurate mass, ion correlations, and isotope ratios (Table 1).

Since we applied an untargeted approach and had no restriction on food and drink consumption, both endogenous and exogenous VOCs were observed. For example, the ions measured at *m*/*z* 81.08, *m*/*z* 137.13, and *m*/*z* 149.10 (Figure S6) tentatively assigned to monoterpene and methyl chavicol (estragole), respectively, are typically of exogenous origin, being extensively used in cuisine and human health care products [23,24]. Therefore, these exogenous compounds were not further investigated in relation to exercise.

**Figure 4:**
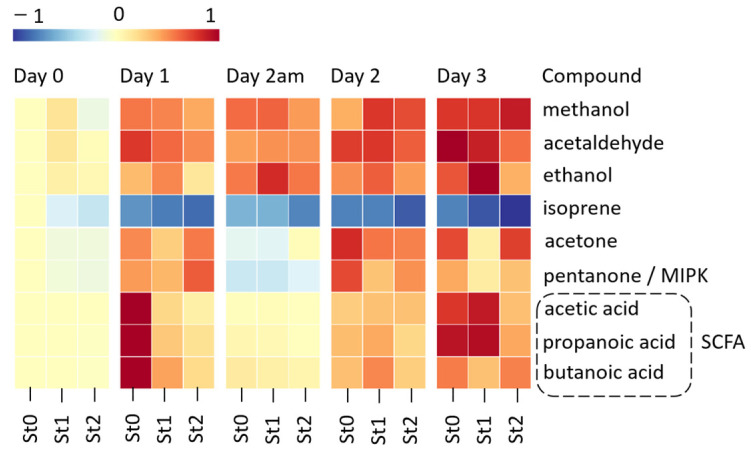
Relative change in the median concentrations of the significant VOCs in relation to the effect of exercise during the 4 days. The concentrations are relative to baseline measurements of before walking of non-statin users (St0) and range scaled by row. All the VOCs, including the short-chain fatty acids (SCFA), reflect a recovery type effect for all participants, by increasing following Day 1 of exercise and then subsequently decreasing after a rest period (Day 2 am), and increasing again after exercise (Day 2, Day 3). Isoprene is an exception, and it shows the opposite trend, i.e., decreasing in relation to exercise.

The next step was to find how the concentration of the endogenous compounds changed in relation to the repeated and prolonged exercise. The relative changes of the median concentrations related to the effect of exercise over 4 days (Day 0–Day 3) for compounds listed in Table 1 are visualized in a heat map (Figure 4). The concentrations are centered on baseline measurements of before walking (Day 0) of non-statin users (St0) and range scaled.

All the above-mentioned endogenous compounds showed a significant change in concentration after exercise compared against the baseline for each of the three groups (Kruskal–Wallis: *p* < 0.001). Most of the VOCs, including acetone, ethanol, acetaldehyde, and methanol, showed a recovery type effect by the participants after the first day of walking, since their concentration in breath increased in relation to exercise (Day 1), and then, it decreased after a period of rest (Day 2 am), which was followed by subsequent increases (Day 2 and Day 3). Interestingly, isoprene showed this trend in the opposite direction: it decreases in relation to exercise, followed by an increase with rest.

A cluster of short-chain fatty acids (SCFA) represented by acetic acid, butanoic acid, and propionic acid, respectively, also displayed this recovery effect (i.e., their concentration changed due to a period of rest). We focused on further comparison between the groups and the effect of exercise and refer to the SCFAs as a cluster. The results were also valid for each acid individually.

**Figure 5:**
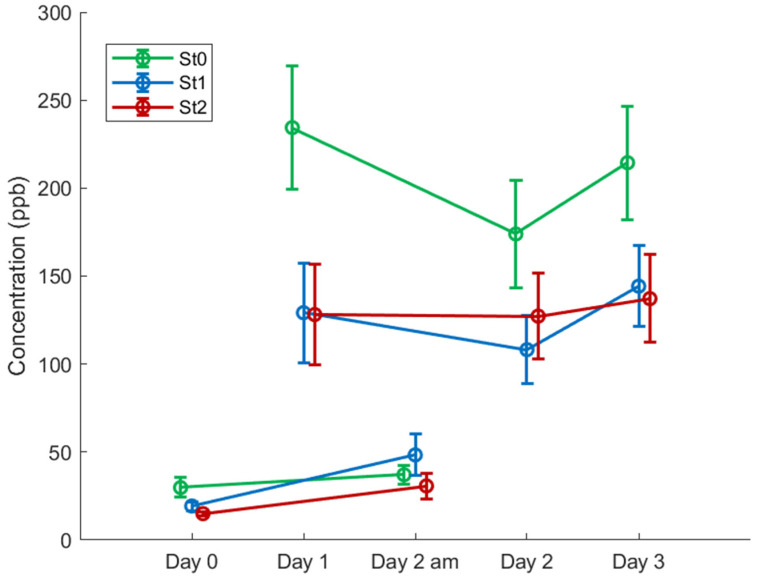
Changes in the concentration of the short-chain fatty acids (SCFAs) cluster over days for each group. Exercise (walking) induced an increase (*p* < 0.001) in SCFAs for all the groups (Day 1, Day 2, and Day 3) compared to (Day 0 and Day 2 am). The SCFAs levels were higher for the non-statin group (St0) compared to statin users (St1: *p* = 0.013 and St2: *p* = 0.005). No difference between St1 and St2 over the post-walking period (*p* = 0.2843). For St0, the SCFAs concentration reached baseline level (*p* = 0.08), whilst for St1 (*p* = 0.009) and St2 (*p* = 0.007), the SCFAs concentration was elevated after a period of rest compared to Day 0. Concentration values are presented as mean ± standard error.

It was found that the total SCFAs concentration did not follow a normal distribution (Lilliefort’s test: *p* < 0.001), and therefore, non-parametric statistical analysis (Kruskal–Wallis test) was applied. The SCFAs concentration was significantly affected by exercise and increased in all the groups as compared to Day 0 (*p* < 0.001). As indicated in Figure 4, the St0 group presented higher post-walking levels compared to the statin group (*p* = 0.013 vs. St1 and *p* = 0.005 vs. St2, for all post-walking time points) (Figure 5). No difference in SCFAs between the statin groups St1 and St2 was found for post walking (Day 1–3; *p* = 0.2843).

Although St0 had elevated SCFAs in comparison with St1 and St2 after the first day of walking, the concentration on Day 2 am reached the same level as on Day 0 (*p* = 0.08). However, the statin users displayed higher concentrations on Day 2 am than on Day 0 (*p* = 0.009 for St1 and *p* = 0.007 for St2, respectively).

## 3. Discussion

### 3.1. Effect of Prolonged Exercise on Breath VOCs

Prolonged exercise is known to induce physiological changes [25,26,27]. Our study revealed a number of endogenous compounds that showed significant changes in concentration over time. We postulate that the primary reason for these changes is due to the effects of exercise.

Endogenous VOCs provide a plethora of information regarding the effect of exercise on various metabolic processes. Several of the presented compounds which changed in relation to exercise have well-known origins, such as acetone and isoprene.

Exhaled acetone is a marker for fatty acid metabolism [28] and indicator of energy balance [13]. It follows that with prolonged exercise, an increase in lipid oxidation will occur, and therefore, an increase in exhaled acetone concentration will be observed. This is indeed reflected in our results. We compared the trend in acetone concentration change from this study with a previous one carried out in 2012 during the same event [13]. We found that in both studies, the acetone concentrations follow the same general trend, i.e., the concentration increases after the first day of walking (Day 1); then, it decreases with rest (Day 2 am), followed by an increase after exercise (Day 2 and 3) (Figure S7). During the current study, breath samples could only be collected at one pre-walking time point (Day 2 am), whereas in the 2012 study, pre-walking and post-walking samples were collected at every time point. Having pre- and post-walking measurements at each time point would have allowed a much more detailed analysis of the recovery effect.

The 2012 study was focused solely on acetone using PTR-MS with an offline sampling protocol, whereas this study was performed using an PTR-ToF-MS with an online sampling protocol and untargeted analysis of the VOCs. Therefore, the similarity in the trends of acetone between the two studies confirm that it may be used as an indicator for fatty acid metabolism and energy balance. The main advantage of online breath analysis is that data can be acquired in real time. This circumvents potential interfering compounds and analyte losses due to the sampling procedure, such as collecting breath into sampling bags [29,30].

Isoprene is formed via the mevalonic acid pathway of cholesterol biosynthesis [31,32,33]. Herein, a decrease in isoprene concentration due to prolonged exercise has been observed. Previous literature reports that isoprene initially increases with the exercise intensity, which is most likely due to changes in pulmonary gas exchange; then, it returns to baseline levels [10]. King et al. observed that for repeated exercise measurements following a short rest period, the increase in isoprene concentration following the onset of exercise was lower in the follow-up measurements. Two hypotheses regarding this effect were offered. Firstly, changes in the blood concentration were due to a depletion/replenishment of an isoprene buffer tissue, for example fat. Secondly, there were sustained functional changes in the lung, which were most likely due to the recruitment and distension of pulmonary capillaries during exercise [10]. In a subsequent publication by King et al. in relation to the endogenous production of breath isoprene, it was postulated that breath isoprene dynamics during physical activity reflect an increased stimulus of a peripheral isoprene source from working muscles [34]. Therefore, it is interesting to note that the overall concentration of isoprene decreased over time during the course of the walking period. Caution is advised when comparing these results directly, as the trend of isoprene decreasing presented in this study is collected at the end of prolonged exercise through walking, whereas King et al. present real-time changes in relation to intense work on an ergometer.

Karl et al. showed that statin use led to a decrease in serum cholesterol and a subsequent parallel decrease in breath isoprene [35]. Thus, it was concluded that the analysis of breath isoprene might serve as a non-invasive tool for monitoring blood cholesterol levels.

The cluster of SCFAs has been found to be responsible for differences between the non-statin and statin users after exercise. They are produced from dietary fiber by intestinal bacteria reflecting a small part of the gut microbiota [10] and represent over 90% of SCFAs in the intestine. During our 2012 study, we measured acetic acid in the headspace of urine samples and found that it increased during exercise [20], with the same trend being reflected in the breath profiles of participants from this study (Figure S8). Acetic acid formation is a result of fatty acid metabolism. It converts into acetyl-CoA (acetyl coenzyme A) and participates in the Krebs cycle, or acetoacetate can be formed from two acetate molecules, which further converts into acetone due to decarboxylation [20]. Butanoic acid is a short-chain fatty acid produced in the colon as the result of carbohydrate fermentation from dietary fiber [36]. During rest, both carbohydrate and lipids are oxidized to provide the energy required to maintain basal metabolic processes in skeletal muscle. During exercise, the need for energy increases significantly; therefore, an increase in the rate of metabolic pathways that oxidize both lipids and carbohydrates occurs [37]. As carbohydrate oxidation is expected to increase during exercise, this may be related to the observed change in concentration of butanoic acid in relation to prolonged exercise, along with the possibility of consuming more carbohydrates from the diet during the prolonged exercise. Nevertheless, exercise can change the human gut microbial composition and associated metabolites independent of diet [38].

Statins have an anti-inflammatory effect [39], and prolonged exercise is known to induce inflammation [14,40]. A previous study indicated that butanoic acid could be related to inflammation or bacterial overgrowth in the gut [41]. Recent findings showed that butanoic acid is produced by the Bacteroides 2 (Bact2), which is a gut enterotype that is associated with inflammation. The study discovered that the use of statin may have a beneficial effect on the bacterial flora by changing gut prevalence of Bact2 [42]. We postulate that this may be an additional reason for the difference observed between the groups and suggest that butanoic acid particularly could be a potential non-invasive marker for monitoring the effect of statin therapy on the gut microbiome.

A potential source for the methanol and ethanol found in exhaled breath is the microbial fermentation of the carbohydrates in the gastro-intestinal tract [43,44]. These compounds may also have an exogenous origin. Typically, the ethanol concentration in breath is significantly increased after the consumption of alcohol or sweet drinks/food when these products have been consumed within 2 h of the breath test [45]. A number of participants consumed alcohol prior to breath sampling, which was reflected in the subsequently acquired breath VOC profiles. In general, three participants were found to be outliers for the ethanol concentration for each post-walking sample time point. Although the statistical analysis pointed out that differences observed are not due to these outliers (alcohol consumption), we had no records of the sugar-containing products consumed by the participants. Therefore, assigning the metabolic origin of the ethanol recorded in breath with certainty is difficult.

### 3.2. Sampling Methodology

We reported an online breath sampling and analysis protocol using PTR-ToF-MS and demonstrated its suitability for real-time measurements in field conditions. Under typical experimental settings, various factors can be controlled, and protocols may be adjusted as the situation dictates. The strength of this study lies in the development and application of a reliable methodology for breath analysis without controlling external factors such as nutrition restriction and yet gathering biologically relevant distinctions.

This approach allowed a high throughput of samples with participants being able to give duplicate breath samples in approximately 2 min. The breath collection procedure was robust (as indicated by the good agreement between duplicates; Figure S1), contamination-free, easy to follow, and without discomfort to the participants.

One of the benefits of the sampling approach developed in our laboratory was the coupling of the PTR-ToF-MS inlet to a commercial breath sampler, so that each participant could monitor their own exhalation flow. Through the implementation of a CO_2_ trigger, we were able to aid our data processing steps by ensuring the extraction of the end-tidal breath fraction for all participants. These steps, amongst others described earlier, guaranteed a reliable and reproducible sampling protocol for the online breath sampling using PTR-ToF-MS.

We postulate non-invasive monitoring of changes in gut microbiome activity. Additional research is needed to investigate in depth the effect of medication and exercise reflected on breath volatiles, through designed studies under controlled conditions (e.g., controlled diet).

## 4. Materials and Methods

### 4.1. Subjects

Sixty-two participants were included, comprising three groups: the non-statin users control group, St0 (*n* = 24), statin users without muscle complaints, St1 (*n* = 17), and statin users with muscle complaints, St2 (*n* = 21). Subjects’ demographic data are listed in Table 2.

### 4.2. Breath Sampling Set-Up and Protocol

**Figure 6:**
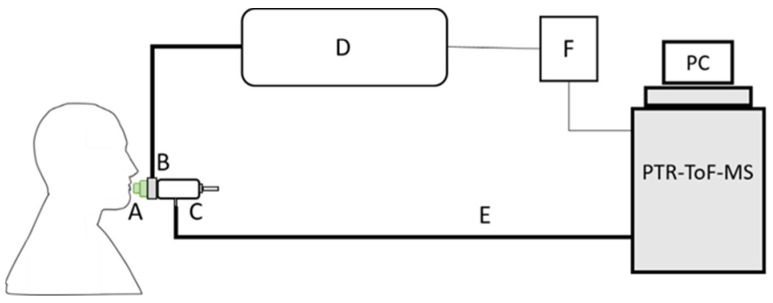
Schematic diagram of the breath sampling setup. Participants exhaled through a bacterial mouth filter (**A**) connected to a CO_2_ sensor (**B**) into a heated buffer pipe (**C**) at a constant flow of 50 ml/sec following a visual indicator on the screen of the breath sampler (**D**) for as long as was comfortable. The pipe is connected to the proton transfer reaction time-of-flight mass spectrometry (PTR-ToF-MS) via a heated sampling line (**E**). When the CO_2_ concentration reached 4%, a CO_2_-flow monitoring trigger signal (**F**) was sent to the PTR-ToF-MS to aid with data processing.

Breath samples were collected at the following time points: pre-walking baseline split over the two days prior to the start of the event (Day 0), post-walking (Day 1, Day 2, and Day 3), Day 2 pre-walking (Day 2 am). Participants provided two online complete, single breath samples at each of these time points via a mouthpiece bacterial filter (GVS, Morecambe, UK) fitted to a heated open-end pipe of a commercial breath sampler (Loccioni^®^, Angeli di Rosora, Italy). We aimed to collect the end-tidal fraction of the breath profile; this was achieved by implementing a CO_2_-controlled sampling protocol. The breath sampler is equipped with a CO_2_ sensor and calibrated buffer pipe, thus allowing for continuous monitoring of the airway pressure and CO_2_ concentration during exhalation. In addition to CO_2_ monitoring, as some of the VOCs are exhalation flow dependent, the participants were asked to maintain an exhalation flow of 50 mL/s for as long as was comfortable per exhalation. The CO_2_ concentration and airway pressure profiles are displayed in a graphical form on the screen of the breath sampler, enabling the participants to visualize their exhalation flow. When the CO_2_ concentration reached 4%, an electrical trigger signal was sent from the breath sampler to the PTR-ToF-MS via a home-made printed circuit board. A schematic diagram of the breath sampling setup is shown in Figure 6. The sampling line was heated to 40 °C to avoid condensation of water vapors from exhaled breath.

This field study did not admit restrictions on food or drink to be placed on participants. Since these participants were enrolled into a multiple testing pipeline (i.e., anthropometric measures, blood sampling, etc.), they were asked to report to the breath sampling station prior to the other testing stations upon completion of the walk each day and on Day 2 before walking.

### 4.3. PTR-ToF-MS Real-Time Measurements of Breath VOCs

Breath samples were measured continuously by a PTR-ToF-MS (model PTR-ToF8000, Ionicon Analytik GmbH, Innsbruck, Austria). Details of PTR-ToF-MS have been extensively reported in the literature [18,46]. Briefly, the instrument consists of an ionization section (the drift tube) where the exhaled VOCs are chemically ionized by proton transfer reaction from hydronium ions (H_3_O^+^) and the detection section where the ionized molecules are separated by their time-of-flight and further detected. The hydronium ions are generated through the electron ionization of water and subsequent ion-molecule reactions with water. This process takes place if the proton affinity of the VOC is higher than that of water (691 kJ mol^−1^).

The continuous side-stream mode of sampling at a flow of 24 mL/min was performed via a 2 m long heated (at 75 °C) ¼″ Silcosteel^®^ transfer-line connected to the breath sampler pipeline. The drift tube temperature and voltage were set to 60 °C and 600 V respectively with an operating pressure of 2.6 mbar, resulting in a reduced electric field (E/N) of 120 Td.

### 4.4. Sampling Reproducibility

To investigate the repeatability of the online sampling, Lin’s concordance correlation coefficient (*R_c_*) was calculated for all duplicate breath samples [15]. The use of *R_c_* has been previously reported for investigating repeatability between breath samples [47]. Lin’s concordance correlation coefficient is a measure to assess the agreement of replicate measurements; the closer *R_c_* is to 1, the higher the degree of agreement between replicates. It was calculated according to Equation (1) in which *S_YX_* is the covariance between the breath samples duplicate, $\bar{X}$ and $\bar{Y}$ represent the average of the breath concentrations for both replicates, and $S_{Y}^{2}$ and $S_{X}^{2}$ are the variance for both replicates. Values for *R_c_* > 0.9 are considered to be excellent in agreement, values between 0.6 and 0.9 are satisfactory in agreement, and *R_c_* < 0.6 unsatisfactory in agreement [16].

(1) $$R_{c}=\frac{2S_{YX}}{{\left(\bar{Y}-\bar{X}\right)}^{2}+S_{Y}^{2}+S_{X}^{2}}$$

### 4.5. PTR-ToF-MS Data Processing

The preprocessing of the raw PTR-ToF-MS data, comprising mass alignment and the integration of manually selected peaks was done using PTR Viewer (v3.2.8, Ionicon Analytik GmbH, Austria). All individual data files containing multiple breath samples were merged using the automation center on the PTR-Viewer software. Mass calibration was performed using the primary ion signal measured via the H_3_^18^O^+^ isotopologue at *m*/*z* 21.02, water-cluster ion H_2_O·H_3_O^+^ at *m*/*z* 39.03, and protonated acetone (C_3_H_6_OH^+^) at *m*/*z* 59.05, respectively. A table of ions was created using multiple peak detection mode with a threshold of 0.1 counts per second. The VOCs were measured in counts per seconds (cps) and normalized to the primary ions, H_3_O^+^ and its water cluster ^18^O isotope *m*/*z* 21.02 (×500) and *m*/*z* 39.03 (×250), respectively.

From this, concentrations were calculated based on the first-order kinetic reaction theoretical equation proposed by Lindinger et al. [48] and de Gouw et al. [49] considering the reaction rate coefficients *k* between VOC and H_3_O^+^ (experimentally determined for specific VOCs such as acetone, isoprene, methanol) [50] and assuming *k* = 2 × 10^−9^ for all other compounds [46]. This approach is particularly useful for the quantification of short-chain fatty acids, for which dynamic calibration methods are technically difficult to perform [51].

The CO_2_ trigger was used as a tracer for breath cycles and to aid in the identification of the end-tidal fraction of breath. A custom “breath tracer” algorithm (MATLAB 2018b, The Mathworks, Inc., Natick, MA, USA) has been internally developed to extract the end-tidal fraction of the breath profile for all participants starting 5 s following from the activation of the 4% CO_2_-flow monitoring trigger and averaging the ion signal recorded over the next 5 s period to improve the signal quality. A complete ToF spectrum ranging from *m*/*z* 20 to 300 was recorded every second. Therefore, 5 spectra have been measured during the 5 s end-tidal fraction considered per subject.

One full exhalation typically lasted a minimum of 20 s. This integration time has been chosen after analyzing the duration of all the breath samples to ensure that the same fraction of exhaled breath can be extracted for all participants.

Hydrocarbons-free air obtained from the room air filtered through a catalyzer (Sensor Sense, Nijmegen, The Netherlands) was used for the baseline measurements in between the breath samples.

The limit of detection (LoD) of the instrument was determined using the instrumental background signal obtained with catalyzed air measurements, following International Union of Pure and Applied Chemistry (IUPAC) guidelines [52], and values below LoD were replaced by LoD value itself [53]. Ions with a concentration higher than the LoD in more than 50% of the breath samples for each of the three groups (St0, St1, and St2) were considered. Based on these criteria, 66 ions were selected for further statistical analysis.

### 4.6. PTR-ToF-MS Compounds Identification

Molecular formulae can be retrieved directly from recorded *m/z* values due to the high mass resolution of the instrument (4000–5000 Δm/m). In several cases, the identification is straightforward, as only one major ion contributes to the measured signal, e.g., acetone, isoprene, propionic acid, methanol–water cluster. The identification of related ions (fragments or clusters) was carried out by Pearson correlation, considering a cut-off value of r^2^ = 0.85. The tentative assignment of VOCs was attributed to corresponding *m/z* values based on these methods of identification and the reported literature on breath research [18]. The identification of the SCFAs was confirmed with the use of standards. As such, compound names have been used throughout rather than *m/z* values alone.

### 4.7. Statistical Analysis

Unsupervised and supervised multivariate methods were applied to investigate the effect of exercise and statin use on the breath VOCs profile.

Multilevel statistical methods (adapted from R package, mixOmics [54]) were used to account for the repeated measurements of the participants over time, with each participant’s Day 0 measurement acting as their own control. Initially, all data were pre-processed by log transformation and mean centering. A multilevel principal component analysis (M-PCA) [55] was carried out as an exploratory data analysis. Each data point was labeled with the time point and the statin group for that individual.

Following this, the supervised methods were used to identify which compounds reflected the effect of prolonged exercise followed by how the concentration of these compounds changed in relation to the exercise. Multilevel partial least squares discriminant analysis (M-PLS-DA) models were created to describe the effect of exercise reflected on the breath profile. Analogous to M-PCA, M-PLS-DA first decomposes the data into between- and within-subject variation, and then, it applies PLS-DA to construct a new set of components (i.e., latent variables, LVs) that have the highest correlation with the groups that are being modeled. Separate M-PLS-DA models were made for each group (St0, St1, and St2), using the following sample time points Day 0, Day 1, Day 2 am, Day 2, and Day 3. Additional models using only the post-walking measurements at Day 1, Day 2, and Day 3, respectively for all three groups were made to further evaluate the effect of exercise repetition. The models were validated using a 5-fold cross-validation with 10 repetitions. Compounds with a Variable Importance in the Projection score greater than 1 were considered significant to the model [56].

The VOCs selected from M-PLS-DA were subjected to further univariate statistical analysis to assess exercise- and statin use-induced changes. Lilliefort’s test was used to determine the normality of the data. Based on the test results, either parametric (ANOVA) or non-parametric statistical analysis (Kruskal–Wallis) was used followed by Tukey’s post hoc correction.

## 5. Conclusions

In this study, we demonstrated an online breath sampling protocol and analysis of exhaled breath using PTR-ToF-MS for use in a field campaign. Despite no restrictions regarding the consumption of food and drinks on the participants, and only 2 min sampling time, the methodology proved to be robust and reliable. Most of the exhaled compounds reflecting the effect of exercise showed a trend of increasing in relation to exercise, except for isoprene. A cluster of SCFAs was identified as the primary cause for the difference in the breath profiles between non-statin and statin users after exercise.

We have provided novel information regarding the use of breathomics as a non-invasive tool for monitoring the changes of human metabolism in relation to exercise and drug therapy, such as statin. In a broader context, this practical approach will become particularly useful for high-throughput screening, prior application of specific testing strategies requiring laboratory support.

## Figures and Tables

**Table 1:** Endogenous compounds related to the differences in the response to exercise and/or the statin use. The m/z values given in parentheses indicate product ion fragments or hydrate clusters.

*m*/*z*	Product Ion Formula	Assigned Compounds	Reference
33.03	CH_4_OH^+^	Methanol	[17]
45.03	C_2_H_4_OH^+^	Acetaldehyde	[18]
47.05	C_2_H_6_OH^+^	Ethanol	[5]
59.05	C_3_H_6_OH^+^	Acetone	[18]
69.07 (41.04)	C_5_H_8_H^+^ (C_3_H_4_H^+^)	Isoprene	[18]
87.09	C_5_H_10_OH^+^	Pentanone/3-methyl-2-butanone	[19]
61.03 (43.02, 79.05)	C_2_H_4_O_2_H^+^ (C_2_H_2_OH^+^, C_2_H_6_O_3_H^+^)	Acetic acid	[18,20,21]
75.05 (57.04, 93.05)	C_3_H_6_O_2_H^+^ C_3_H_4_OH^+^ C_3_H_8_O_3_H^+^	Propanoic acid	[10,18,21]
89.06 (71.05)	C_4_H_8_O_2_H^+^ C_4_H_6_OH^+^	Butanoic acid	[18,21,22]

**Table 2:** Demographic data of the study participants.

Characteristic	Non-Statin Users (St0) (*n* = 24)	Statin Users without Muscle Complaints (St1) (*n* = 17)	Statin Users with Muscle Complaints (St2) (*n* = 17)
Age (years; mean ± SD)	66 ± 6	66 ± 6	62 ± 7
Sex (F/M)	7/17	4/13	7/14
Walking distance per day (30/40 km)	17/7	10/7	11/10
Body mass index (BMI; kg/m^2^; mean ± SD)	26.5 ± 3.8	26.7 ± 3.2	26.1 ± 3.3

## Data Availability

The data presented in this study are available on request from the corresponding author.

## Supplementary Materials

The following are available online at https://www.mdpi.com/2218-1989/11/4/192/s1, Figure S1: Assessment of intra-participant variability through the calculation of Lin’s concordance correlation coefficient, Figure S2, Influence of exogenous sources on several participants duplicate breath samples collected one after another, separated by the dashed boxes, Figure S3: Multilevel PCA scores plot for the effect of walking and statin use on the breath VOC profile, Figure S4: M-PLS-DA models for statin users for all sampling time points, Figure S5: M-PLS-DA model for statin users for all post-walking measurements, Figure S6: Venn diagram of ions with discriminative ions for each M-PLS-DA model, Figure S7: Comparison of breath acetone concentrations for non-statin users in the current study and urine headspace acetone of the healthy volunteers during the Four Days Marches 2012, Figure S8: Acetic acid in breath (current study) and from urine headspace (2012 study) for healthy participants in the Four Days marches.
