# Supplementary material for: Exhaled Breath Reflects Prolonged Exercise and Statin Use during a Field Campaign
Source: Metabolites. 2021 Mar 24;11(4):192. doi: 10.3390/metabo11040192 (PMC8064097; doi:10.3390/metabo11040192)
Supplement: Supplementary file 1 [file metabolites-11-00192-s001.pdf]

Supplementary material

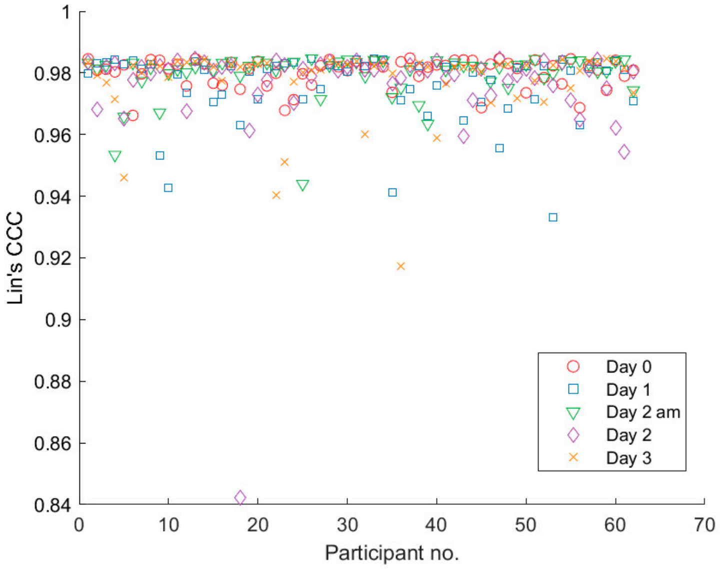

Figure S1. Assessment of intra-participant variability through calculation of Lin's concordance correlation coefficient. Excellent repeatability, as indicated by a Lin's concordance coefficient, R_c, >0.9 was observed for all duplicate samples, except for one participant (R_c = 0.84).

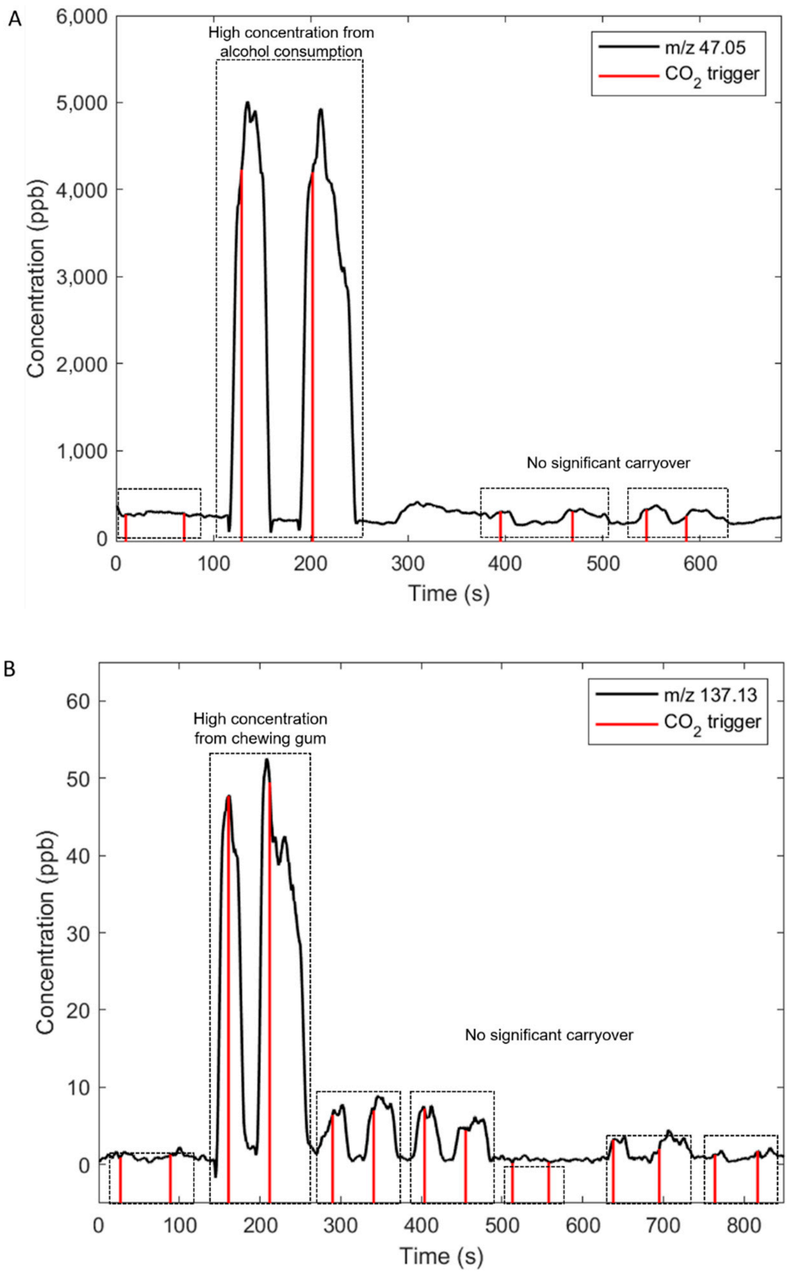

Figure S2. Influence of exogenous sources on several participants duplicate breath samples collected one after another, separated by the dashed boxes. The data in **A** and **B** were collected at different time points and are from different participants. High concentrations of **A**) m/z 47.07 (ethanol) from one participant, (2nd out of 4) and **B)** m/z 137.13 (monoterpene), from another participant (2nd out of 6) sampled during the pipeline flow. A manual flushing procedure used between breath samples was sufficient to minimise carryover effects, even with high concentration signals originating from exogenous sources.

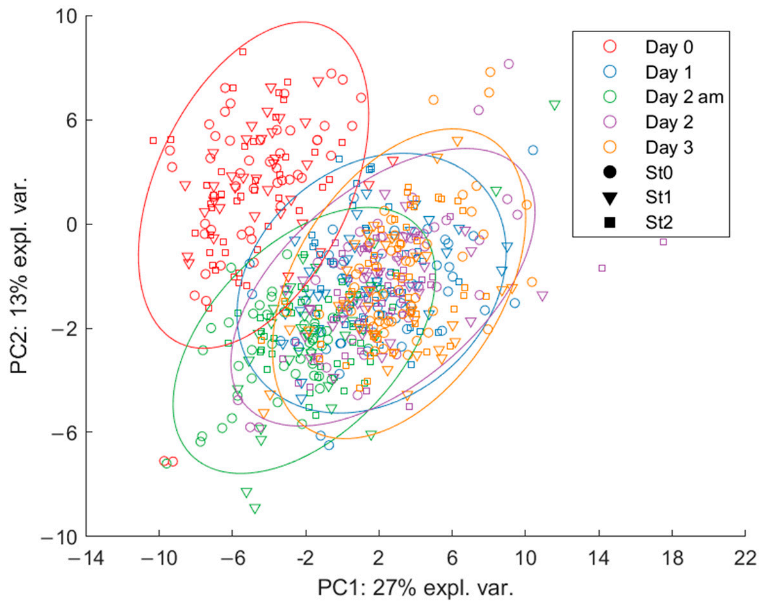

Figure S3. Multilevel PCA scores plot for the effect of walking and statin use on the breath VOC profile. The difference between Day 0 before walking and the other time points indicates that the breath profile changes following the onset of prolonged exercise. There is no clear clustering based on statin use.

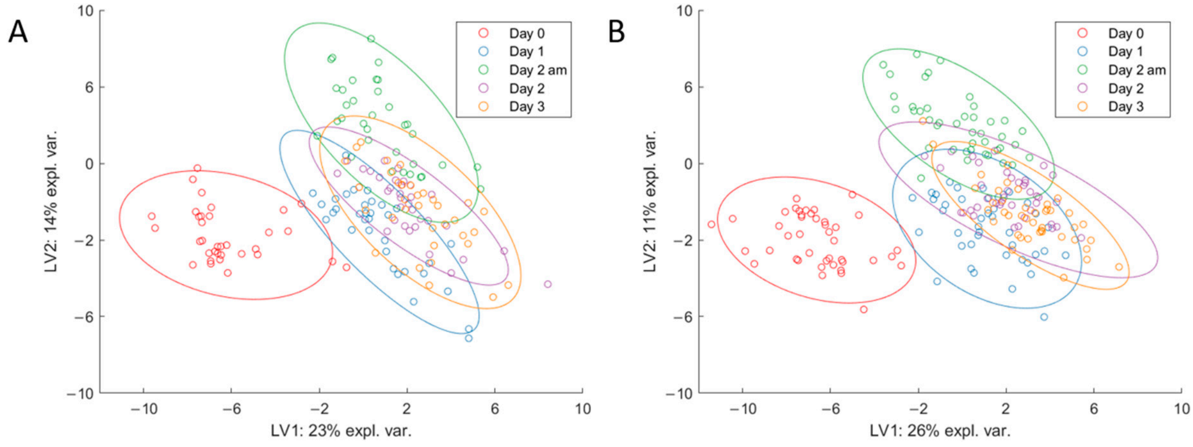

Figure S4. M-PLS-DA models for Statin users St1 (A) and St2 (B), respectively, including all sample time points. Similar to the non-statin users (St0), clear separation between Day 0 and the remaining time points is present, indicating a significant change in the breath profile at the onset of exercise compared against the Day 0 baseline measurement.

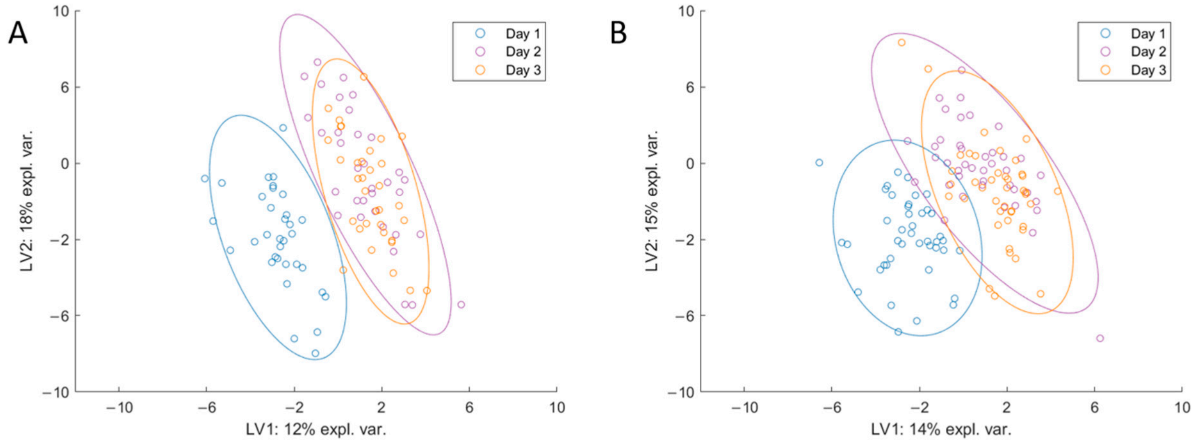

Figure S5. M-PLS-DA model for St1 (A) and St2 (B) for all post walking measurements (Day 1, Day 2 and Day 3). There is a difference in clustering of the groups. There is high overlap between Day 2 and Day 3 however there is a degree of separation between Day 1 and the other time points. These results suggest Day 2 and Day 3 breath profiles are more similar than Day 1, indicating a possible adaptation to steady state over the course of strenuous walking over the week.

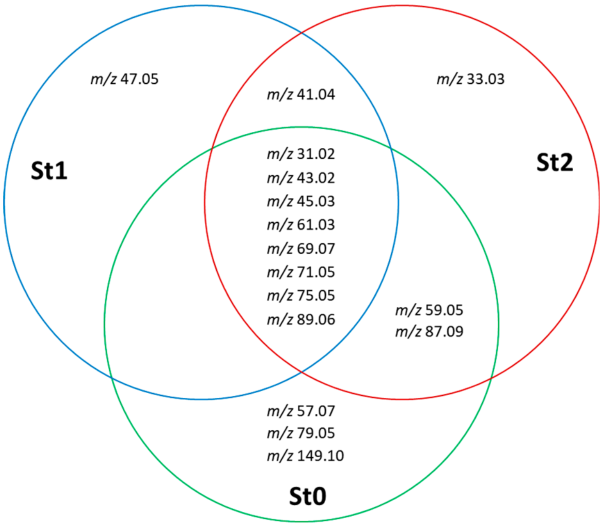

Figure S6. Venn diagram of ions with VIP scores > 1 for each M-PLS-DA model, showing discriminative and common ions between the groups. The ions in the middle section were significant in all three models designed for the St0, St1 and St2, respectively, and a small number of ions were significant in one of the three models only, highlighting that there are potential differences in the response to exercise based on the statin use. Tentative assignment of these ions to specific compounds is given in Table 1 of the manuscript.

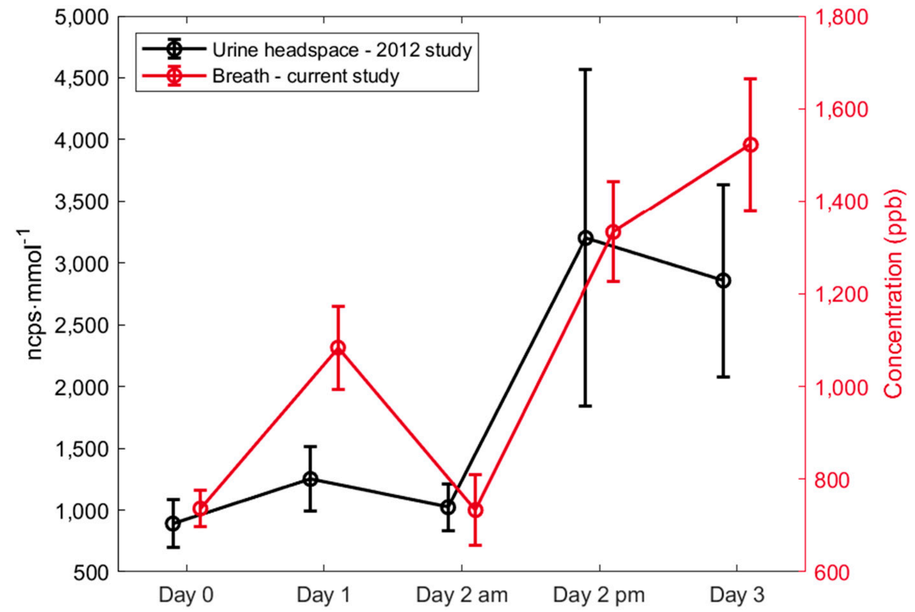

Figure S7. Comparison of breath acetone concentrations for St0 participants of this study and urine headspace acetone of the healthy volunteers during the Four Days Marches 2012. Urine headspace concentrations were normalized against creatinine Acetone concentration follows similar trends in relation to exercise by increasing after the 1st day of walking (Day1), then decreases with rest (Day 2 am), followed by an increase after exercise (Day 2 and 3).

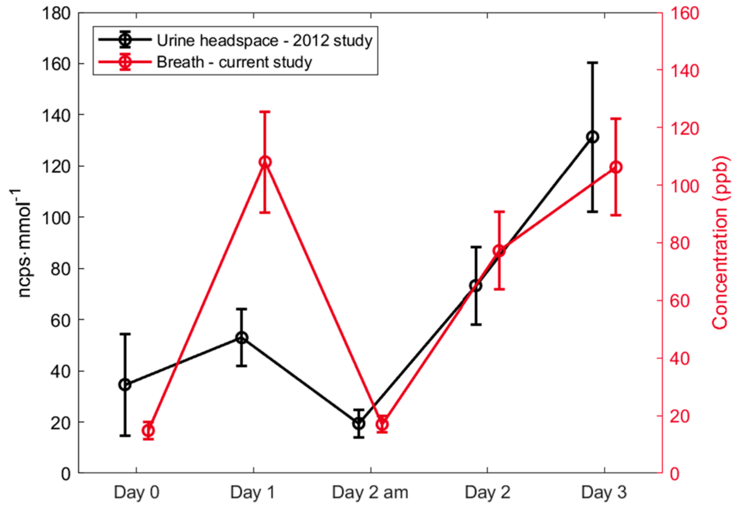

Comparison (urine headspace)	p-value
Day 0 vs. Day 1	$p < 0.05$
Day 1 vs. Day 2 am	$p < 0.05$
Day 2 am vs. Day 2	$p < 0.05$
Day 2 vs. Day 3	ns

Figure S7. Acetic acid in breath (current study) and from urine headspace (2012 study) for healthy participants in the 4 Days marches. Urine headspace concentrations were normalized against creatinine. Similar trend observed in both studies. For both studies, the results are presented as mean concentrations ± standard error. Statistical analysis was carried using Kruskal-Wallis test.
